# Numerical Simulation of Failure Behavior of Reinforced Concrete Shear Walls by a Micropolar Peridynamic Model

**DOI:** 10.3390/ma16083199

**Published:** 2023-04-18

**Authors:** Feng Shen, Zihan Chen, Jia Zheng, Qing Zhang

**Affiliations:** 1 School of Civil Engineering, Suzhou University of Science and Technology, Suzhou 215011, China; chenzihan1998@126.com; 2 Suzhou Jcon Greenbuild Fabricated Co., Ltd., Suzhou 215004, China; zhengjia1996912@163.com; 3 Department of Engineering Mechanics, Hohai University, Nanjing 211100, China; lxzhangqing@hhu.edu.cn

**Keywords:** peridynamics, improved micropolar model, RC shear walls, numerical simulation, impact failure

## Abstract

A reinforced concrete shear wall is an important building structure. Once damage occurs, it not only causes great losses to various properties but also seriously endangers people’s lives. It is difficult to achieve an accurate description of the damage process using the traditional numerical calculation method, which is based on the continuous medium theory. Its bottleneck lies in the crack-induced discontinuity, whereas the adopted numerical analysis method has the continuity requirement. The peridynamic theory can solve discontinuity problems and analyze material damage processes during crack expansion. In this paper, the quasi-static failure and impact failure of shear walls are simulated by improved micropolar peridynamics, which provides the whole process of microdefect growth, damage accumulation, crack initiation, and propagation. The peridynamic predictions are in good match with the current experiment observations, filling the gap of shear wall failure behavior in existing research.

## 1. Introduction

Reinforced concrete (RC) shear walls are widely used in concrete structures for their low cost and high stiffness advantages. However, RC shear walls have deficiencies such as poor ductility, early cracking, and apparent shear damage, which limit their use to some extent. As the study of the damage behavior of RC shear walls mainly focused on two aspects of seismic performance study and plane impact resistance of shear walls, Jiang et al. [1] analyzed the law of influence of axial compression ratio on the seismic capacity and damage development of an RC shear wall. Su et al. [2] proposed a two-parameter seismic damage model based on a combination of stiffness degradation and deformation. Lopes et al. [3,4] proposed a new method for calculating the shear bearing capacity by conducting repeated loading tests on low RC shear walls. Paulay et al. [5] proposed that a proper arrangement of diagonal reinforcement in making low RC shear walls obtain a better energy dissipation effect and achieve bending damage. Lefas et al. [6] proposed that the shear capacity of RC shear walls is related to the triaxial compressive stress conditions generated in the bottom section of the compressive zone. Sittipunt et al. [7] considered the effect of loading mechanisms on RC shear wall experiments. They proposed a finite element model that can reflect the performance of RC shear wall members with different reinforcement under reciprocal loading and their possible damage patterns. Vallenas et al. [8] proposed an analytical model for high RC shear walls under monotonic loading because shear deformation occupies a relatively large proportion of high RC shear walls. Terzioglu et al. [9] studied the effect of shear, bending, and slip deformation on the lateral deformation of RC shear walls. Salonikios et al. [10,11] introduced a theoretical formula for the ultimate strength of RC shear walls and proposed a method for calculating the plastic hinge length of RC shear wall members with small aspect ratios. Looi et al. [12] found that axial loading significantly affects crack distribution, failure modes, and deformation properties and proposed two improved empirical prediction models. Houshmand-Sarvestani et al. [13] found that a steel plate with added damping and stiffness (ADAS) dampers can improve the ductility of the steel shear walls in addition to dissipating energy. Yi et al. [14] designed a pendulum tester with adjustable hammer weight and speed to perform out-of-plane impact tests on RC shear walls. Tan et al. [15] discussed the cyclic shear performance of the buckling-restrained cold-formed steel plate shear wall (SPSW) under different buckling constraints. Zhang et al. [16] predicted the seismic performance of shear walls through mechanical learning (ML) methods, and these have a high accuracy in predicting shear wall failure patterns, and predicting the shear strength and flexural strength of reinforced concrete walls more accurately and effectively than existing design formulas.

In order to explore the damage mechanism of shear walls, it is necessary to describe their damage process and damage pattern under load accurately. The finite element method (FEM) is the main tool for the numerical simulation of concrete structures. Simple modeling and fast operation. However, the finite element method is based on the theory of continuous media. Hence the calculation of cracks in the structure will deviate from reality. Extended finite element (X-FEM) is a major improvement in the finite element method [17]. The calculation grid used is not related to the internal composition of the structure. There is no need to perform a grid weight division many times. Therefore, crack expansion simulation can be performed. However, it is still challenging to accurately and efficiently model complex cracking problems, such as three-dimensional composite crack growth problems with random tracks or complex loads. Discrete elements (DEM) solve the problem of discontinuity by dividing the structure into many elements and defining the bonding constitutive between the elements [18]. However, its data structure is complex, and how to distinguish adjacent elements, and the contact determination of the element will make the calculation very complicated. It is difficult to achieve an accurate description of the damage process using the traditional numerical calculation method, which is based on the continuous medium theory. Its bottleneck lies in the crack-induced discontinuity, whereas the adopted numerical analysis method has the continuity requirement. This difficulty is expected to be solved with the emergence of the peridynamic theory. The peridynamic theory is a set of theories no longer based on the traditional continuity assumption and local contact action. The peridynamic theory uses spatial integration and time-differential equations to describe the interaction of masses and solves the crack sprouting and extension problem by equations of motion in integral form, which fundamentally avoids the singularity caused by discontinuity problems and eliminates the need to introduce additional fracture criteria. By introducing the concept of damage to the material and judging the cumulative number of bond fractures to describe the formation and extension of cracks, it enables cracks to emerge, extend and branch naturally in the object. Thus, it has obvious advantages in the study of structural damage problems, and many excellent research results have emerged.

In 2000, Silling et al. [19] proposed the peridynamics theory and algorithms, demonstrating their unique advantages in solving damage problems in solid materials and structures and providing a promising theoretical framework for developing robust numerical models. Gerstle et al. [20,21] have improved based on the PD theory based on “bond”. Based on the radial movement of material points, the relative rotation of the material point pairs is considered, and the micropolar peridynamic model is proposed. Zhang et al. [22] proposed a new bond-slip model and demonstrated its powerful ability to simulate the damage behavior of reinforced concrete structures. Yaghoobi et al. [23,24] introduced the semi-discrete method into the micropolar PD model to study the effect of reinforcing fibers on the analysis of cracks in cementitious materials. Shi et al. [25] proposed homogeneous and heterogeneous bonding for the same and different materials of adjacent material points. Zheng et al. [26] improved the micropolar peridynamics model by considering concrete materials’ low tensile strength and high compressive strength and weakened the interaction between rebar and concrete in reinforced concrete structures. Zhang et al. [27] and Zheng et al. [28] coupled PD with an atomistic (AM) model and classical continuum mechanics (CCM) model, respectively, which guaranteed accuracy and enhanced computational efficiency. Shen et al. [29] established a new hybrid model of peridynamics (PD) and finite element method (FEM) based on implicit schemes to improve the computational efficiency. This paper focuses on the failure behavior of reinforced concrete walls under lateral loading and impact loading through a reasonable modeling approach and improved principal structure, providing an effective method for solving the shear wall force damage problem in engineering practice.

The remaining parts of this article are as follows: Section 2 summarizes the basic theory of peridynamics, improved micropolar peridynamic model, and modeling technique. Section 3 introduces the numerical implementation of the explicit dynamic algorithm. The proposed method provides two examples to verify its reliability, including the damage of the reinforced concrete shear wall under the static action in Section 4. It also includes data compared with experiments. For the conclusion, see Section 5.

## 2. Basic Theory of Peridynamics

### 2.1. Basic Ideas

The peridynamic theory was introduced by Silling [19] in 2000, which can be used at different scales, from micro to macro, does not assume continuous or small deformation behavior, and has no requirement for the concepts of stress and strain. The peridynamic equation of motion is an integral formulation rather than a partial differential equation, which faces many difficulties at discontinuities. Internal forces are expressed through nonlocal interactions between pairs of material points, and damage can be regarded as a part of the constitutive model. The peridynamic model allows discontinuities and cracks to emerge naturally.

**Figure 1:**
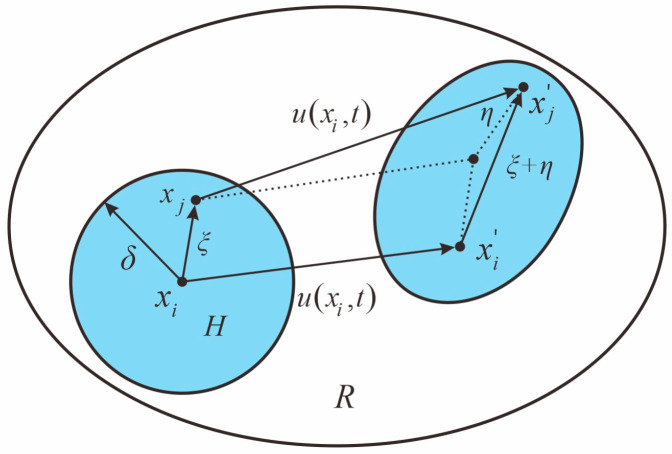
Interaction between material points.

As shown in Figure 1, the object in the spatial domain R is uniformly discretized into material points with material information, and at a certain moment t, each material point $x_{i}$ does not interact only with its neighboring material points, but there is an intrinsic force of equal magnitude and opposite direction force between all other material points in its near-field range H, which can be obtained as

(1) $$\rho \frac{\partial^{2}u\left(x_{i},t\right)}{\partial t^{2}}=\int_{H}f\left(u\left(x_{j},t\right)-u\left(x_{i},t\right),x_{j}-x_{i}\right)dV_{x_{j}}+b\left(x_{i},t\right)$$

where ρ is the material point material density, u is the displacement of the material point, f is the interaction force between the two material points, $\partial^{2}u\left(x_{i},t\right)/\partial t^{2}$ is the acceleration, and b is the prescribed external body force density of the material point $x_{i}$.

(2) $$\xi =x_{j}-x_{i},\eta =u\left(x_{j},t\right)-u\left(x_{i},t\right)$$

where ξ is the length of the bond, and η is the relative displacement of the bond; the expression is as follows:

The interaction force f can be written as

(3) $$f\left(\xi ,\eta \right)=\frac{\left(\xi +\eta \right)}{∥\xi +\eta ∥}c\left(\xi \right)s\mu \left(\xi ,t\right)$$

where c(ξ) is a micromodulus function of the bond, and the relative stretch s of the bond can be expressed as

(4) $$s=\frac{∥\xi +\eta ∥-∥\xi ∥}{∥\xi ∥}$$

Represents the historically related scalar value function between the connection status of the bond between the material points, μ is given as follows:

(5) $$\mu \left(\xi ,t\right)=\left\{ \begin{array}{ll} \;1, & s(\xi ,t^{\prime })<s_{0}\left(t\right)\;\mathrm{for}\;\mathrm{all}\;0\leq t^{\prime }\leq t\\ \;0, & \mathrm{otherwise} \end{array}\right.$$

where $s_{0}$ is a critical stretch of the material for failure. Failure occurs when the stretch between two particles exceeds the critical stretch, and these two particles stop interacting with each other. Even though S is referred to as a particle property, bond breaking must be symmetric for all pairs of particles that share a bond. The elastic modulus E, tensile strength $f_{t}$, and compressive strength $f_{c}$ can be used to express the critical stretch of concrete material [26] as follows:

(6) $$\left\{ \begin{array}{ll} \;s_{t}=0.8f_{t}/E, & s(\xi )>0\\ \;s_{c}=-f_{c}/E, & s(\xi )<0 \end{array}\right.$$

When the stretch s between material points exceeds the critical stretch $s_{t}$, it means that the bond between them is broken.

The broken condition of the bonds to the rest material points in the horizon determines the damage to the material point. This method is closer to the determination of real damage. The inexplicit nature of local damage at a particle arising from the introduction of failure in the constitutive model, an unambiguous notion of local damage at a point is described as a scalar-valued function φ(x,t):

(7) $$\varphi \left(x,t\right)=1-\frac{\int_{H}\mu \left(x,\xi ,t\right)dH}{\int_{H}dH}$$

where H is the neighborhood of the particle x, which is assumed to be a spherical region centered at x with the radius of the material horizon, and is analogous to the cutoff radius used in molecular dynamics.

The function φ(x,t) reflects the degree of damage, φ∈[0,1]. φ=0 indicates that the material point is undamaged whereas φ=1 means the material point has been completely damaged.

### 2.2. Micropolar Peridynamics

The microscopic elastic-brittle model (PMB) based on the “bond” theory only considers the radial movement between the material points and thus has a Poisson’s ratio limitation. To solve this problem, the radial movement and rotation of the material points are considered simultaneously, and the equation of motion is expressed as

(8) $$\left\{ \begin{array}{l} \;\rho \frac{\partial^{2}u\left(x_{i},t\right)}{\partial t^{2}}=\int_{H}f\left(u\left(x_{j},t\right)-u\left(x_{i},t\right),x_{j}-x_{i},\theta_{i},\theta_{j}\right)dV_{x_{j}}+b\left(x_{i},t\right)\\ \;I\frac{\partial^{2}\theta \left(x_{i},t\right)}{\partial t^{2}}=\int_{H}m\left(u\left(x_{j},t\right)-u\left(x_{i},t\right),x_{j}-x_{i},\theta_{i},\theta_{j}\right)dV_{x_{j}}+M\left(x_{i},t\right) \end{array}\right.$$

where I denotes the rotational moment of inertia, $\theta_{i}$, $\theta_{j}$ denotes the rotational displacement of the material point, m is the moment of the material points, and M is the external moment.

The force function between the material point pairs f is related to the displacement u between the material points by the expression

(9) f=ku

Equation (9) can also be represented in the form of a matrix

(10) $$\left(\begin{array}{l} f_{xi}\\ f_{yi}\\ m_{zi}\\ f_{xj}\\ f_{yj}\\ m_{zj} \end{array}\right)=\left(\begin{array}{cccccc} EA/\xi & 0 & 0 & -EA/\xi & 0 & 0\\ 0 & 12EI/\xi^{3} & 6EI/\xi^{2} & 0 & -12EI/\xi^{3} & 6EI/\xi^{2}\\ 0 & 6EI/\xi^{2} & 4EI/\xi & 0 & -6EI/\xi^{2} & 2EI/\xi \\ -EA/\xi & 0 & 0 & EA/\xi & 0 & 0\\ 0 & -12EI/\xi^{3} & -6EI/\xi^{2} & 0 & 12EI/\xi^{3} & -6EI/\xi^{2}\\ 0 & 6EI/\xi^{2} & 2EI/\xi & 0 & -6EI/\xi^{2} & 4EI/\xi \end{array}\right)\left(\begin{array}{l} u_{i}\\ v_{i}\\ \theta_{zi}\\ u_{j}\\ v_{j}\\ \theta_{zj} \end{array}\right)$$

where EA=c, EI=d, c is the tensile micromodulus, and d is the bending micromodulus of the bond. I is the rotational inertia, and E is the elastic modulus.

Considering the material point pair between the remote force action, after correction to obtain the tensile and bending micromodulus coefficients in the case of plane stress problem are as follows [30]:

(11) $$\{\begin{array}{c} c=\frac{105E}{4\pi \delta^{3}(1-v)}{\left(1-{\left(\frac{\xi }{\delta }\right)}^{2}\right)}^{2}\\ d=\frac{5E(1-3\nu )}{16\pi \delta (1-v^{2})}{\left(1-{\left(\frac{\xi }{\delta }\right)}^{2}\right)}^{2} \end{array}$$

The micropolar peridynamics break through the limitation of a certain Poisson’s ratio. The Poisson’s ratio for the plane stress problem is changed from 0.33 to a range of 0–0.33, whereas the Poisson’s ratio for the three-dimensional problem is changed from 0.25 to a range of 0–0.25.

### 2.3. Rebar Constitutive Model

**Figure 2:**
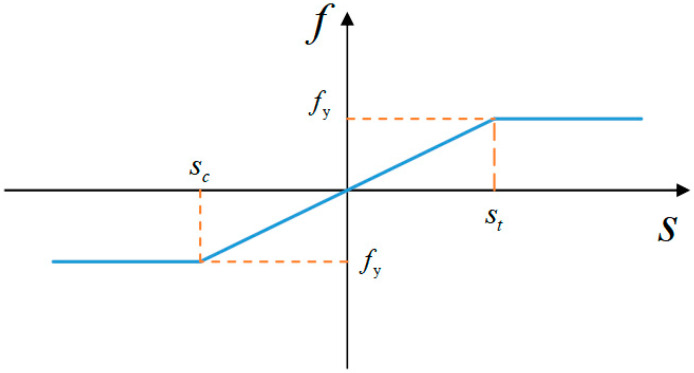
Reinforcement constitutive model.

This article assumes that the rebars in the reinforced concrete structure cannot break due to damage. The rebars use a damage model similar to the ideal elastoplastic model. Their tensile and compressive properties are assumed to be the same. The force between the material points is linearly elastic when the stretch between the material points of the rebars is between the critical stretch and the critical compression rate. The force between the material points is constant when the stretch between the material points exceeds the critical stretch. The horizontal line in the model signifies that the bar has reached yield, as shown in Figure 2.

### 2.4. Concrete Constitutive Model

**Figure 3:**
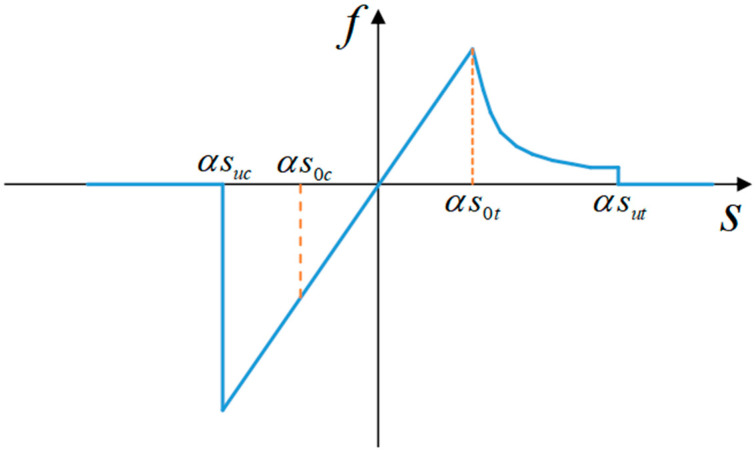
Adhesion-tension relationship for concrete material model.

To describe the damage behavior of concrete, the micropolar force vector-bond stretch relationship is proposed [23], which improves the concrete instanton of the micropolar peridynamic model by introducing a correction factor for bond damage with the following expression: F=(1−λ)KU, λ is a damage factor between 0 and 1. λ=0 means no damage, whereas λ=1 means complete damage. $s_{ft}$ controls the slope of the softening branch. Considering that the compressive strength of concrete is much higher than the tensile strength, concrete will experience fatigue, and its bearing capacity will be reduced when suffering loads in the opposite direction. This paper reduces $s_{uc}$, $s_{c}$, $s_{t}$, and $s_{ut}$ when the direction of the bond force changes, to achieve the role of simulating concrete fatigue. The specific operation is as follows: when the bond force positive and negative sign changes, $s_{uc}$, $s_{0c}$, $s_{0t}$, and $s_{ut}$ will multiply a weakening parameter α, thus reflecting concrete fatigue, concrete is more easily destroyed. As shown in Equation (12) and the bond force-stretch function is shown in Figure 3.

(12) $$f=\{\begin{array}{ll} 1 & (s\text{<}\alpha s_{uc})\\ 0 & \left(\alpha s_{uc}\leq s\leq \alpha s_{0t}\right)\\ 1-\frac{\alpha s_{0t}}{s}e^{-\frac{s-\alpha s_{0t}}{\alpha s_{ft}-\alpha s_{0t}}} & \left(\alpha s_{0t}\leq s\text{<}\alpha s_{ut}\right)\\ 1 & \left(\alpha s_{ut}\leq s\right) \end{array}$$

In the present constitute model, the whole damage of the connection can be expressed in two ways. The bond breaks completely when the bond extension s is less than $\alpha s_{uc}$ or greater than $\alpha s_{ut}$, then the concrete is damaged. When the bond extension s is between $\alpha s_{uc}$ and $\alpha s_{0t}$, the intrinsic force and stretch vary linearly. When the bond extension s is between $\alpha s_{0t}$ and $\alpha s_{ut}$ the intrinsic force decreases exponentially.

### 2.5. Interface Processing

How to describe the interface between steel and concrete is a great challenge in peridynamics. As the strength of the bond at the interface is lower in concrete, this paper adopts a weakened connection property. In reinforced concrete structures, the two materials with different physical properties mainly rely on the bond stress between them to work together. Sliding damage to the reinforced concrete bonding interface is the reason for the performance to failure. Bond failure is a cross-scale process in which a large amount of fine-scale damage develops and induces macroscopic failure [31]. To solve the problem, it should be distinguished from bonds between homogeneous materials. A little plastic deformation begins to occur in concrete, microcracks begin to sprout, and strains grow rapidly faster when the tensile stress σ reaches 40–60% of the peak stress $f_{y}$. Therefore, the interaction between rebar and concrete in reinforced concrete structures is weakened and expressed as follows [32]:

(13) $$\left\{ \begin{array}{l} \;c=\min\left\{c_{x_{i}},0.4\left(c_{x_{i}}+c_{x_{j}}\right),c_{x_{j}}\right\}\\ \;d=\min\left\{d_{x_{i}},0.4\left(d_{x_{i}}+d_{x_{j}}\right),d_{x_{j}}\right\} \end{array}\right.$$

where $c_{x_{i}}$ is the tensile micromodulus, $d_{x_{i}}$ is the bending micromodulus of material point $x_{i}$, $c_{x_{j}}$ is the tensile micromodulus, and $d_{x_{j}}$ is the bending micromodulus of material point $x_{j}$.

## 3. Numerical Implementation

### 3.1. Artificial Damping

According to the basic idea of peridynamics, the object is uniformly discretized into material points, and the material point spacing is taken as Δx. In solving the quasi-static problem, the dynamic relaxation algorithm is borrowed, and artificial damping is added to the equations of motion, and the equations converge rapidly, then the equations are as follows:

(14) $$\{\begin{array}{l} \rho \ddot{u}(x,t)+C\dot{u}(x,t)=\sum_{j=1}^{p}f(\eta ,\xi ,\theta_{i},\theta_{j})V_{j}+b(x,t)\\ I\ddot{\theta }(x,t)+C\dot{\theta }(x,t)=\sum_{j=1}^{p}m(\eta ,\xi ,\theta_{i},\theta_{j})V_{j}+M(x,t) \end{array}$$

where p is the number of other material points in the horizon, and $V_{j}$ is the volume of material point j. C is the artificial damping, whose size only affects the speed of computational convergence.

### 3.2. Impact Contact Algorithm

**Figure 4:**
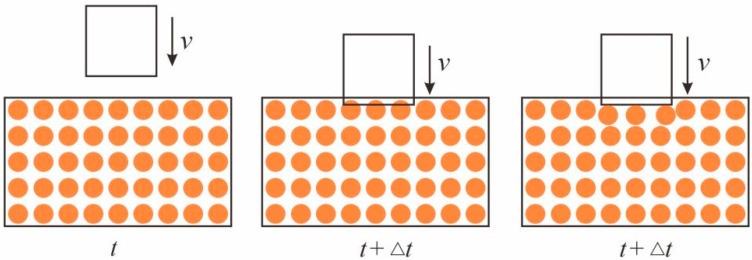
Rigid projectile impact contact model.

As shown in Figure 4, the impacted concrete beam is stationary when the falling hammer first starts to contact the concrete beam. The combined force at each material point is zero. When the falling hammer penetrates the concrete beam, the material point occupied by the falling hammer is squeezed away to the surface of the falling hammer [33].

The coordinate of a material point at time t is $x_{k}$, $u_{\left(k\right)}^{t}$ is the displacement, and $v_{\left(k\right)}^{t}$ is the velocity. The falling hammer is in contact with the concrete beam at moment t, and the material point overlaps with the falling hammer at moment t+Δt. In the process of impact, the falling hammer continuously moved the material points occupied by the concrete beam from its track to the closest position on the surface of the falling hammer, and the new displacement after the displacement is calculated as $u_{\left(k\right)}^{t+\Delta t}$, and the new velocity is ${\bar{v}}_{\left(k\right)}^{t+\Delta t}$:

(15) $${\bar{v}}_{\left(k\right)}^{t+\Delta t}=\frac{{\bar{u}}_{\left(k\right)}^{t+\Delta t}-u_{\left(k\right)}^{t}}{\Delta t}$$

The combined force on the falling hammer is

(16) $$F^{t+\Delta t}=\sum_{k=1}-\rho_{k}\frac{{\bar{v}}_{\left(k\right)}^{t+\Delta t}-v_{\left(k\right)}^{t}}{\Delta t}V_{\left(k\right)}\lambda_{\left(k\right)}^{t+\Delta t}$$

where $\lambda_{\left(k\right)}^{t+\Delta t}=1$ means that the falling hammer and the material point overlap, $\lambda_{\left(k\right)}^{t+\Delta t}=0$ means that the two do not overlap situation.

To prevent the occurrence of mutual penetration deformation between material points when simulating concrete structures, a short-range repulsive force term needs to be added between material points, and the short-range repulsive force equation is as follows:

(17) $$f_{s}\left(y^{\prime },y\right)=\min\left\{0,\frac{135E}{\pi \delta^{4}}\left(\left\|y^{\prime }-y\right\|\right)-d\right\}\frac{y^{\prime }-y}{\left\|y^{\prime }-y\right\|}$$

The expression of the short-range force action distance between material points is written as:

(18) $$d=\min\left\{0.9\left\|x^{\prime }-x\right\|,1.35|\Delta x|\right\}$$

## 4. Numerical Examples

### 4.1. Quasi-Static Damage of RC Shear Walls

**Figure 5:**
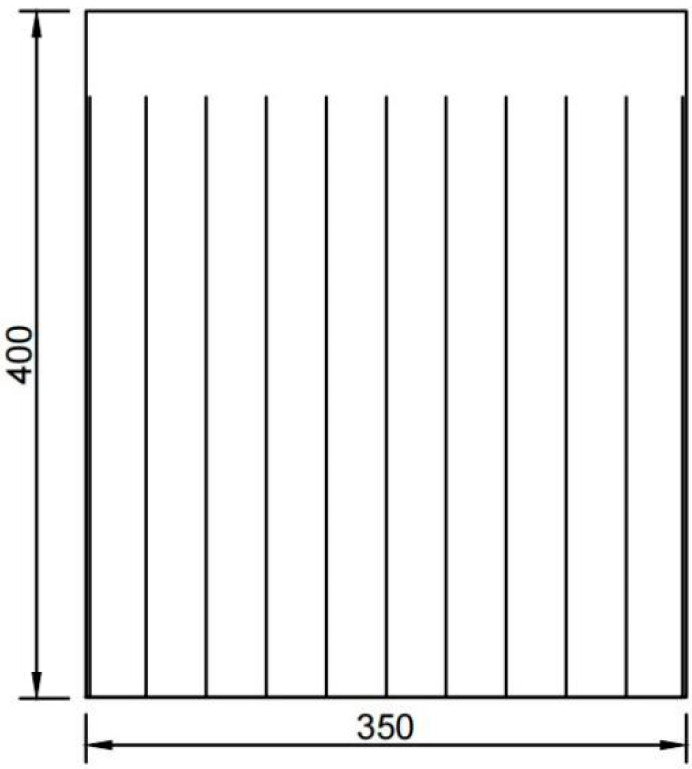
Geometrical dimensions of the RC shear walls.

The RC shear wall is shown in Figure 5. Concrete properties are stated as follows. The concrete strength grade is C30, Young’s modulus *E* = 21.4 GPa, Poisson’s ratio *v* = 0.2, the mass density *ρ* = 2400 kg/m^3^, the tensile strength of 2.94 N/mm^2^, and the compressive strength of 29.1 N/mm^2^. Steel properties are also described as follows. The rebar grade is HRB400, Young’s modulus *E* = 211 GPa, Poisson’s ratio *v* = 0.3, and the mass density *ρ* = 7800 kg/m^3^. The model contains a total of 140,751 cell points with a time step of 10^−7^ s. The loading zone is 350 mm × 50 mm in the upper part, and 350 mm × 350 mm in the lower part for observing displacement and damage. The axial pressure ratio is controlled by applying vertical force in the upper half zone, and for comparison with the experimental data, the axial pressure ratio is controlled to be 0.1, and then lateral force is applied to observe the damage pattern of the RC shear walls.

**Figure 6:**
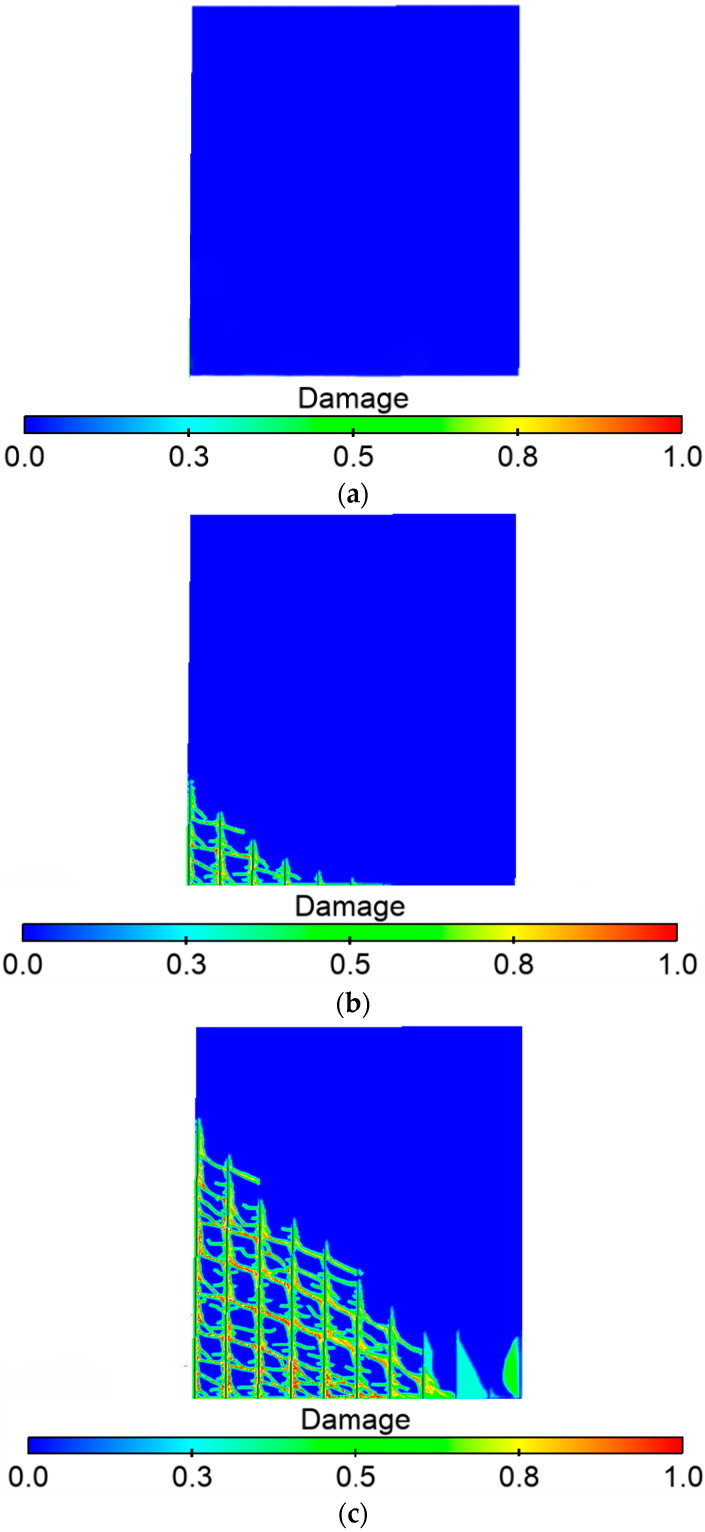

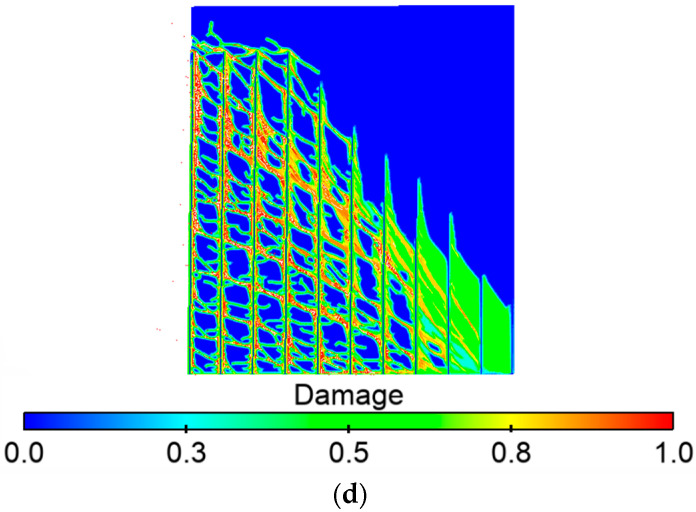
RC shear walls unilateral force damage process. (**a**) Initial stage; (**b**) Crack initiation; (**c**) Crack propagation and (**d**) Structure failure.

Figure 6a–d are the schematic diagrams of the crack expansion during the damage process of the RC shear walls. At first, there is no damage to the member. Then cracks appear in the wall from the bottom with increased applied load. Cracks appear at the bond between bars and concrete and develop rapidly. As the load continues to increase, cracks in the corner of the wall start to develop upward, and significant damage appears on the compressed side. Finally, cracks connecting the diagonal of the shear wall appeared, meanwhile significant damage appeared in the compressed area, and the RC shear walls were damaged.

**Figure 7:**
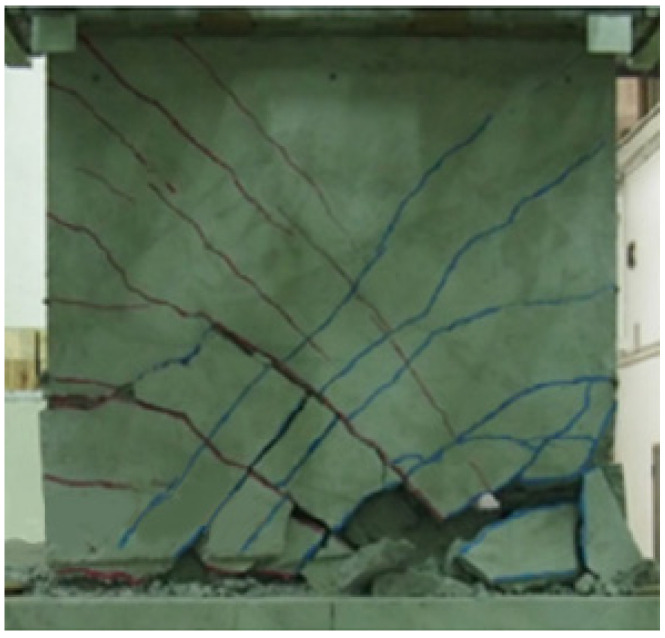
Damage modes of RC shear walls [12]. Copyright 2017, Engineering Structures.

**Figure 8:**
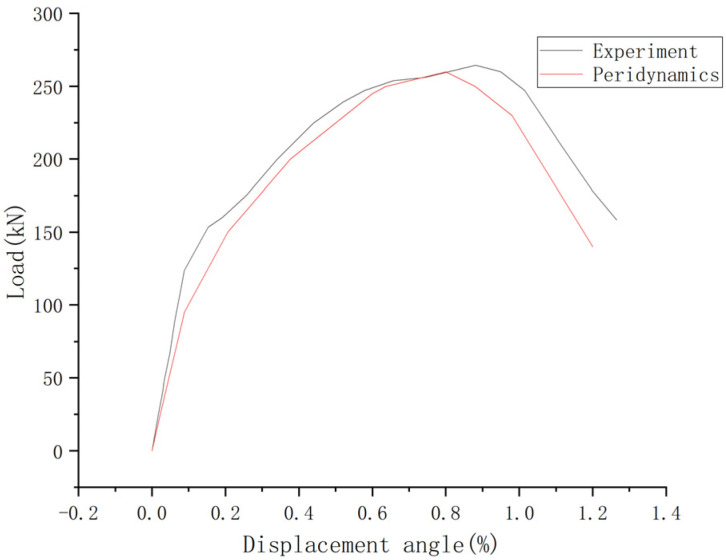
Load-displacement curve [12].

As shown in Figure 7, the rack development and final damage results of the peridynamic are similar to the experimental results [12]. The numerical result of the load-displacement curve is consistent with the experiment as shown in Figure 8.

### 4.2. Failure Behavior of the RC Shear Walls under Impact Loading

**Figure 9:**
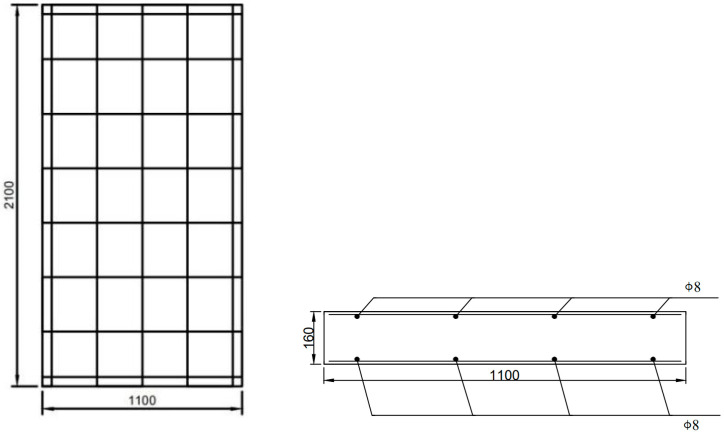
RC shear walls dimensions and reinforcement distribution.

As shown in Figure 9, the RC shear wall’s dimensions are 1.1 m × 2.1 m × 0.16 m. Material properties for concrete are Young’s modulus *E* = 30 GPa, Poisson’s ratio *v* = 0.2, and density *ρ* = 2400 kg/m^3^. The compressive strength of concrete is 30.7 N/mm^2^, which is assumed to be a homogeneous, isotropic, elastic, and brittle material. The properties of the rebars: Young’s modulus *E* = 200 GPa, Poisson’s ratio v = 0.3, yield strength *f_y_* = 442 MPa and mass density *ρ* = 7800 kg/m^3^. The model contains a total of 398,157 cell points with a time step of 10^−7^ s.

A 640 kg drop hammer falls freely from 0.75 m. The impact side of the shear wall is front and the other side is back. The falling hammer is only along the impact direction movement to ensure that the falling hammer in the impact process does not occur in other directions of displacement, to avoid causing the loss of impact energy. The constraint is achieved by fixing the displacement of the two short sides of the RC shear walls.

**Figure 10:**
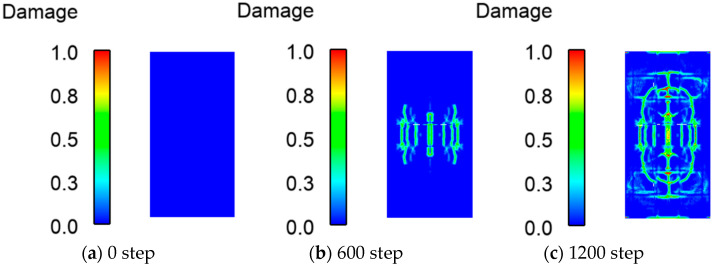

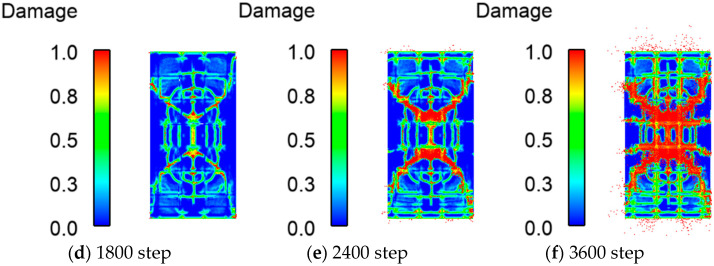
Crack development process at the front of the RC shear wall after falling hammer contact with the RC shear wall.

**Figure 11:**
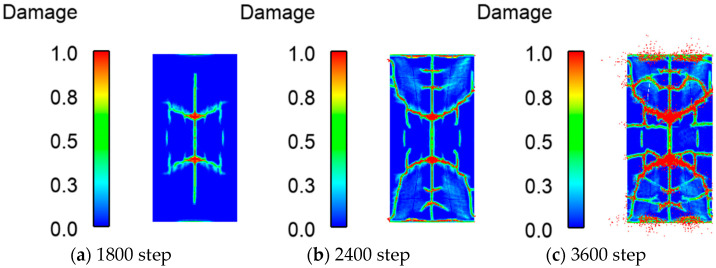
Crack development process at the back of the RC shear wall after falling hammer contact with the RC shear wall.

Figure 10 is a schematic diagram of the crack expansion process on the impacted side. The failure process is shown in Figure 10a–e. During the initial stage of simulation, no microcracks were observed. When the falling hammer contacted the wall, the cracks arose in the center and propagated in all directions. As the damage increased, transverse cracks appeared, and damage grew at the fixed position. As the simulation proceeded, cracks covered the surface. Then the forked cracks on the wall surface deepened, and the damage increased rapidly. Figure 11 shows the crack expansion process on the back side. Compared with the front side, the back-side cracks appear later, and there are fewer cracks and less damage.

**Figure 12:**
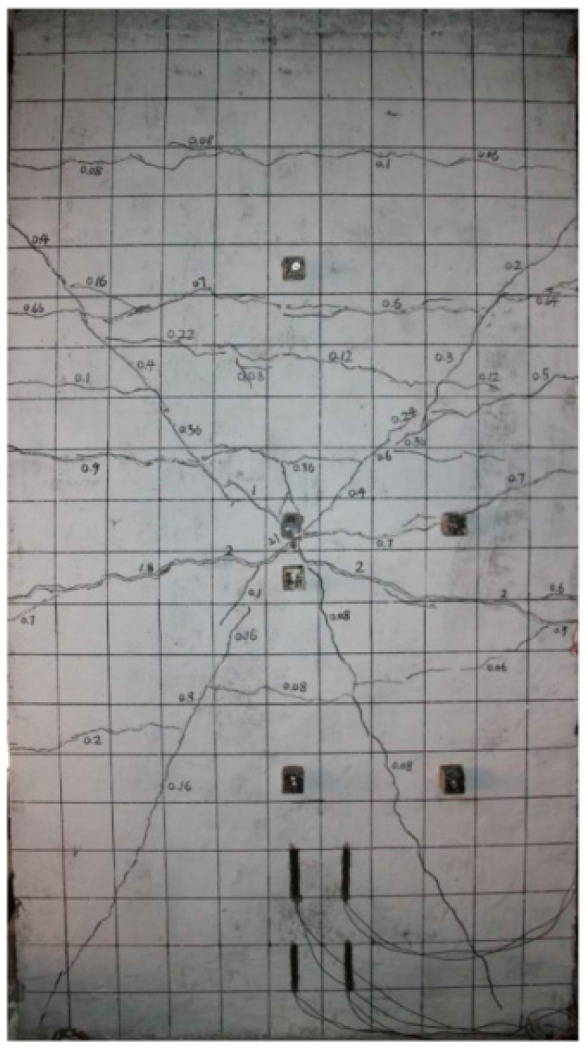
Cracks at the back of the RC shear wall [14]. Copyright 2019, Vibration and Shock.

**Figure 13:**
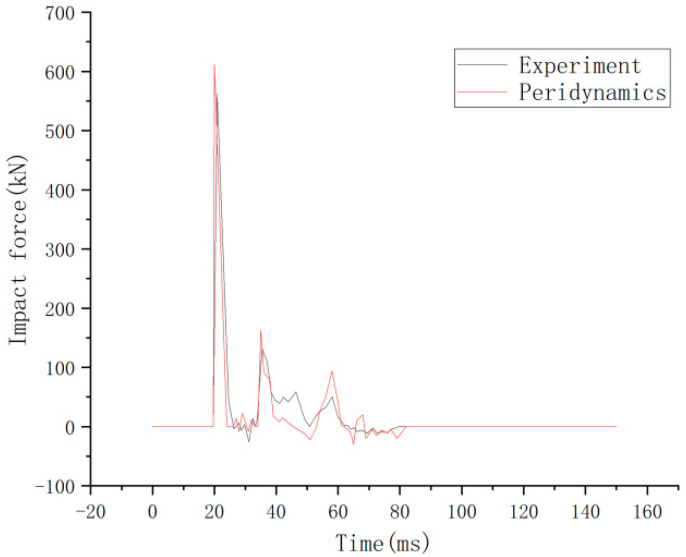
Time histories of impact force [14].

**Figure 14:**
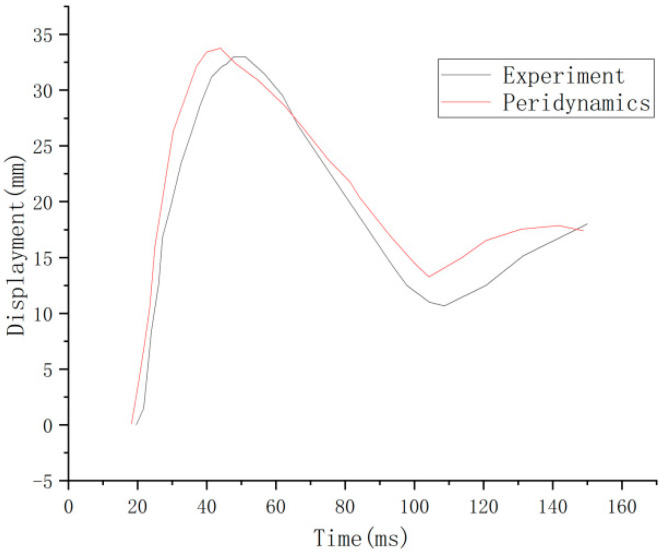
Midpoint displacement of the wall [14].

It is consistent with the crack development pattern of the test results in Figure 12. The impact force-time course curve is shown in Figure 13. The peak test impact force is at 562 kN, and the peridynamic theory simulation result is at 612 kN, with an error of 8.9%. The time displacement curve at the midpoint of the RC shear walls is shown in Figure 14. The maximum displacement of the experimental midpoint is 33 mm, and the peridynamic theory simulation results in 33.8 mm, with an error of 2.4%.

## 5. Conclusions

To accurately describe the failure behavior and damage evolution of reinforced concrete shear walls, this paper improves the micropolar peridynamic model. Considering that the compressive strength of concrete is much higher than the tensile strength and the effect of concrete fatigue on concrete strength, the role of the “bond” between reinforcement and concrete is weakened. Based on the improved micropolar peridynamic, the failure behavior of the quasi-static reinforced concrete shear wall is simulated. The cracks appeared from the bond between the rebars and the concrete in the shear zone and developed rapidly. Finally, cracks connecting the diagonal of the shear wall appeared; meanwhile, significant damage appeared in the compressed area, and the RC shear walls were damaged. Since the reinforcement and concrete are idealized native structures, there is a specific difference in the simulated results and an inevitable error in the load-displacement angle curve.

In addition, through the contact algorithm method, a whole failure process of RC shear walls was acquired, which from cracks appearing from the center of the shear wall, the cracks develop around, transverse cracks appear, and finally, the forked cracks deepen, and lead to the failure of the shear wall. The appearance and development of front and back cracks are consistent with the experiment, the impact displacement curve is well-fitted, and the maximum impact force error is within a small range.

The results show that the peridynamic method has a better ability to simulate the cumulative accumulation and gradual destruction process of RC shear walls, which provides a compelling new method to solve the RC shear walls damage problem in engineering. The improved micropolar peridynamics model is accurate and effective to calculate the failure behavior of RC shear walls. However, the peridynamics has the problems of low computational efficiency and difficult results processing, which need to be further studied.

## Data Availability

Not applicable.
